# 4-Bromo­pyridinium tri­chlorido­stannate(II)

**DOI:** 10.1107/S2414314626008771

**Published:** 2026-08-28

**Authors:** Felix Henkel, Hans Reuter

**Affiliations:** a Chemistry, Osnabrück University, Barabarstr. 7, 49069 Osnabrück, Germany

**Keywords:** crystal structure, tri­chlorido­stannate(II)-ion, 4-bromo­pyridinium, tetrel bonds, π–π inter­action

## Abstract

Single crystals of the title salt, (C_5_H_5_BrN)[SnCl_3_] or [4-BrPyH][SnCl_3_], were prepared from aqueous solutions of 4-bromo­pyridinium hydro­chloride, [4-BrPyH]Cl, and tin dichloride, SnCl_2_. The cation has bond lengths and angles similar to those found in its other salts. In its first coordination sphere, the anion has a trigonal–pyramidal, *tpy*, structure with the Sn^II^ atom at the apex and the three chlorine atoms at the base. The tin–chlorine bond lengths are all greater than 2.5 Å and the bond angles are all less than 92°, which indicates the presence of *tetrel* bonds. These occur along the twofold screw-axis in the *b-*axis direction and extend the coordination sphere of the tin atom to a highly distorted, 3_3_-aaa octa­hedron. In the crystal, the cations are stacked parallel to one another at varying distances along the *a*-axis direction, with weak π–π inter­actions occurring on both sides. Additional N—H⋯Cl hydrogen bonds are bifurcated and weak.

## Abstract 2

The crystal structure of the title compound comprises [Sn^II^Cl_3_]^−^ and [4-BrPyH]^+^ ions. The structural parameters of the anion and the cation are presented and compared with those in other compounds. Inter­molecular inter­actions such as tetrel bonds, π–π inter­actions and N—H⋯Cl hydrogen bonds are analysed.

## Graphical abstract

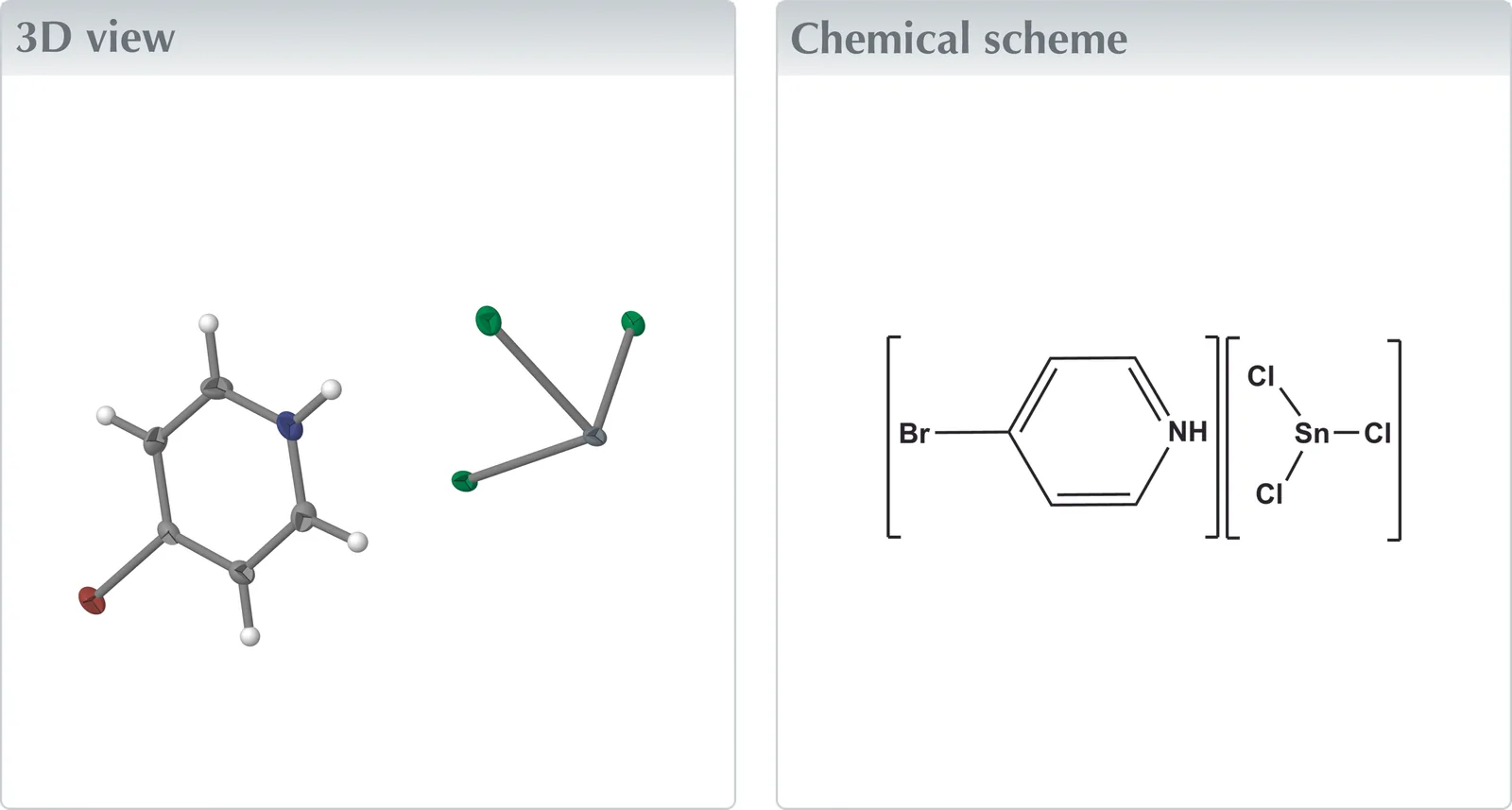

## Structure description

The 4-bromo­pyrinium ion, [4-BrPyH]^+^, is not only a cation frequently used in connection with transition-metal anions such as [CuCl_4_]^2−^ (Willett *et al.*, 2003 ▸), [RuCl_6_]^2−^ (Guthrie *et al.*, 2026 ▸), [FeCl_4_]^−^ (Zordan *et al.*, 2005 ▸) and [CoHal_4_]^−^ (Minguez Espallargas *et al.*, 2006 ▸), but also when it comes to crystallizing anions of low-valent *p*-block elements. Typical examples in this case are anions of trivalent anti­mony, such as [Sb^III^_2_Hal_9_]^3−^ (Nicolas *et al.*, 2022 ▸), trivalent bis­muth, such as [Bi^III^_2_Hal_10_]^4−^ (Adonin *et al.*, 2018 ▸) or tetra­valent tellurium, such as [Te^IV^I_6_]^2−^ (Walusiak *et al.*, 2023 ▸). In this series of compounds, the title compound 4-bromo­pyridinium tri­chlorido­stannate(II), [^4^BrpyH][Sn^II^Cl_3_], is the first to contain an anion of divalent tin.

**Figure 1:**
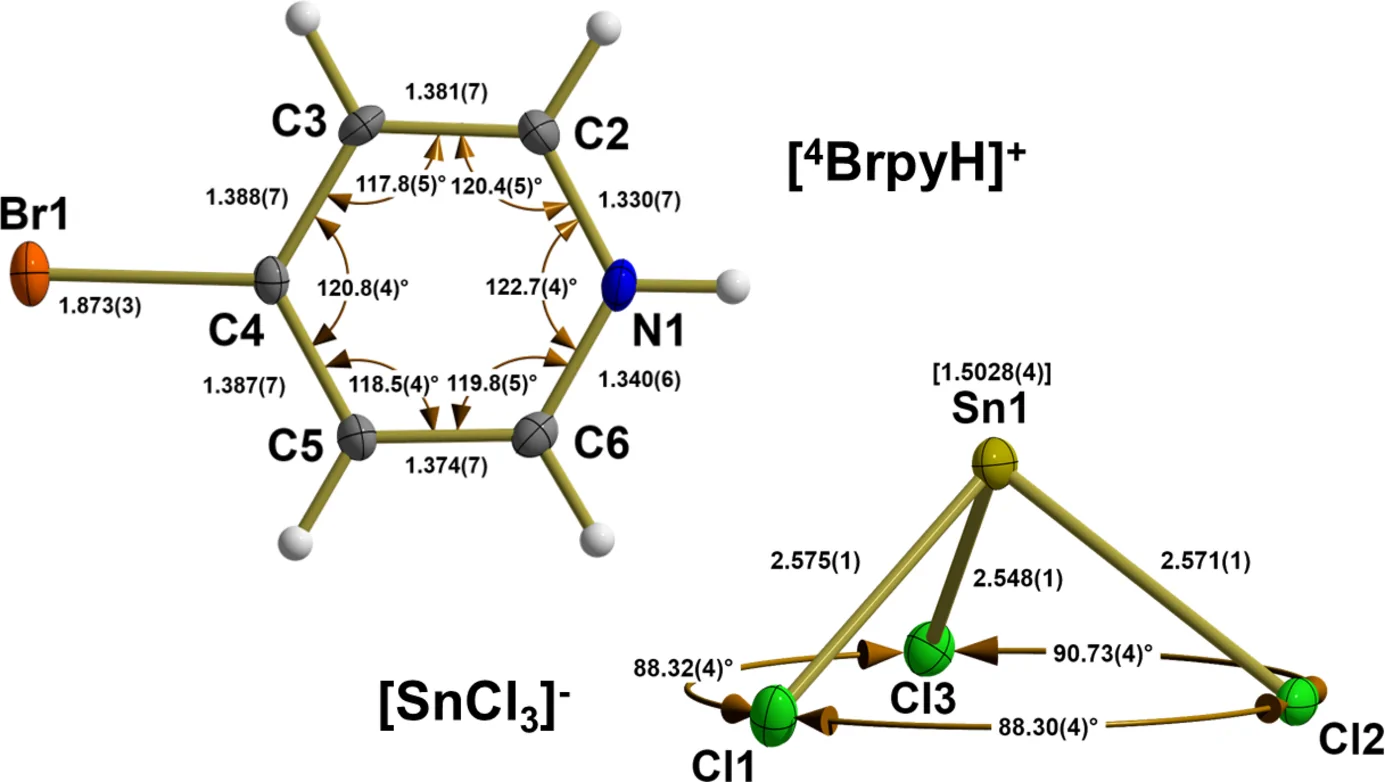
The [4-BrPyH]^+^ and [SnCl_3_]^−^ ion of the asymmetric unit with atom numbering, bond lengths (Å), bond angles (°) and distance of the tin atom from the basal plane in square brackets. With the exception of the hydrogen atoms, which are shown as spheres of arbitrary radius, all other atoms are drawn as anisotropic displacement ellipsoids at the 50% probability level.

The asymmetric unit (Fig. 1 ▸ and Table 1 ▸) comprises one formula unit of each ion with all atoms in general positions. The cation exhibits the well-known structural features evident from the preceding publications: (i) the backbone of the pyridinium ion is nearly planar [Δ = ±0.011 (5) Å], (ii) the bromine atom lies slightly outside [0.0921 (5) Å] this plane, (iii) the bond angle at the nitro­gen atom is significantly widened [122.7 (4)°], (iv) the bond angles at the carbon atoms in the *meta* position relative to the nitro­gen atom are significantly narrowed [117.8 (5)/118.5 (4)°], (v) within the ring, the N—C bond lengths [1.330 (7)/1.340 (6) Å] are shorter than the C—C bonds [mean value: 1.383 (6) Å] and (vi) a Br—C distance of 1.873 (5) Å.

With respect to the first coordination sphere, in which the Sn^II^ atom (Fig. 1 ▸) achieves an electron octet, the [SnCl_3_]^−^ ion exhibits a trigonal–pyramidal, *tpy*, structure, with the tin atom at the apex and the three chlorine atoms in the basal plane. Given bond angles of around 90°, it can be assumed that the three empty, orthogonal 5*p* orbitals of the tin(II) atom are involved in the bonds.

Based on their structural parameters, the tri­chlorido­stannate(II) ions ions can be divided into two groups: in the first group, the tin–chlorine bond lengths are with a few exceptions shorter than 2.50 Å and the bond angles are all greater than 92°. This group includes, for example, the tri­chlorido­stannate(II) ions found in the compounds [Ph_4_P][SnCl_3_] (Müller *et al.*, 1982 ▸), [Sn^II^Cl(18-crown-6)][SnCl_3_] (Drew & Nicholson, 1986 ▸) and [Hf^IV^(O^*t*^Bu)_3_(thf)_3_][SnCl_3_] (Njua *et al.*, 2010 ▸). In the second group, one, two or all three tin–chlorine bonds are longer than 2.50 Å and one or more bond angles are less than 90°. Typical examples are: [MPyrH][SnCl_3_] (MPyrH = 1-methyl­pyrrolidin-1-ium; Liu *et al.*, 2019 ▸), [Li(12-crown-4)(thf)][SnCl3] (Izod *et al.*, 2012 ▸) and [^*t*^BuNH_3_][SnCl_3_] (Veith *et al.*, 1988 ▸). In the first group, these are "naked" ions without strong inter­molecular inter­actions involving the Sn^II^ atom with other building units; in the second group, they are ions in which the Sn^II^ atoms form additional so-called *tetrel bonds* (Bauzá *et al.*, 2019 ▸; Brammer *et al.*, 2023 ▸), characterized by the fact that they occur in a *trans* position to existing strong 2*c*–2*e* bonds. In the broadest sense, *tetrel bonds* can be regarded as extensive 3*c*–4*e* bonds based on an almost linear alignment of the *p*-orbitals of the three atoms involved in the *X*—Sn⋯Y arrangement (Reuter, 2025 ▸).

**Figure 2:**
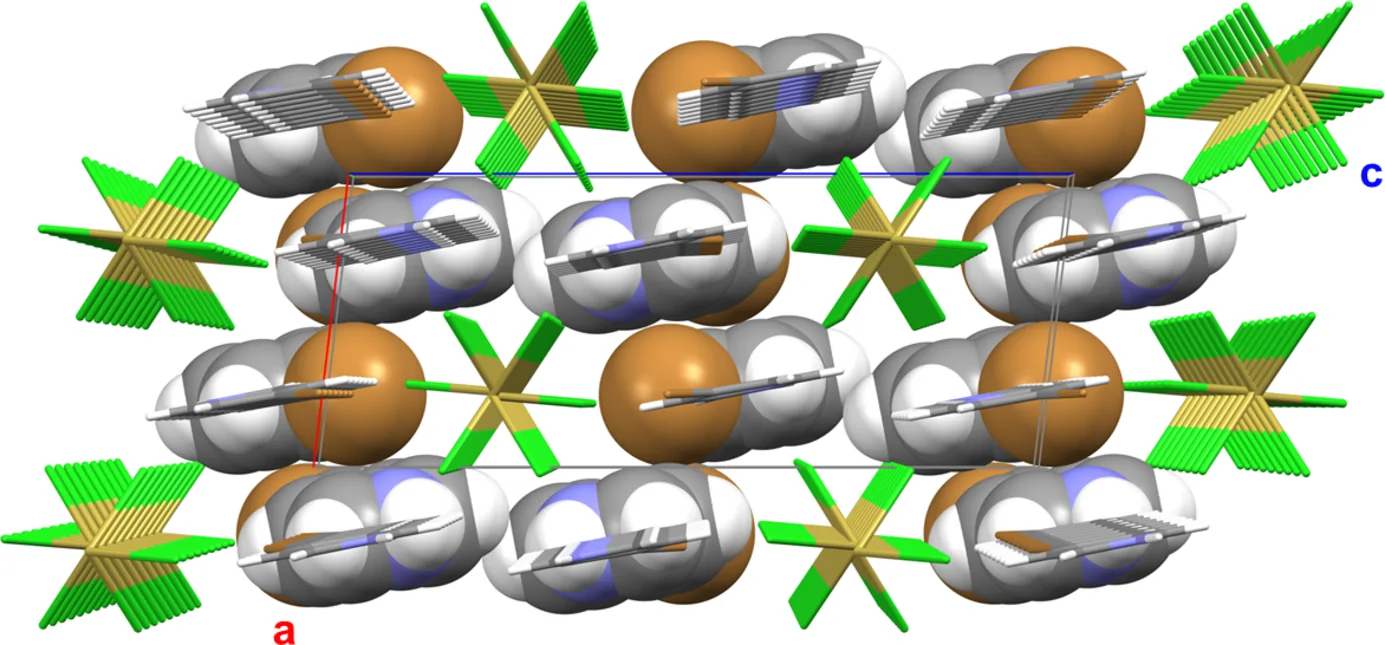
Perspective view into the crystal of [4-BrPyH][SnCl_3_] looking down the *b* axis. All components are drawn as stick models with the cations in the background additionally represented as space-filling models. Colour code and van der Waals radii used: Sn = bronze, Cl = green, C = dark grey, 1.70 Å, H = white, 1.20 Å, N = light- blue, 1.55 Å, Br = brown, 1.85 Å.

**Figure 3:**
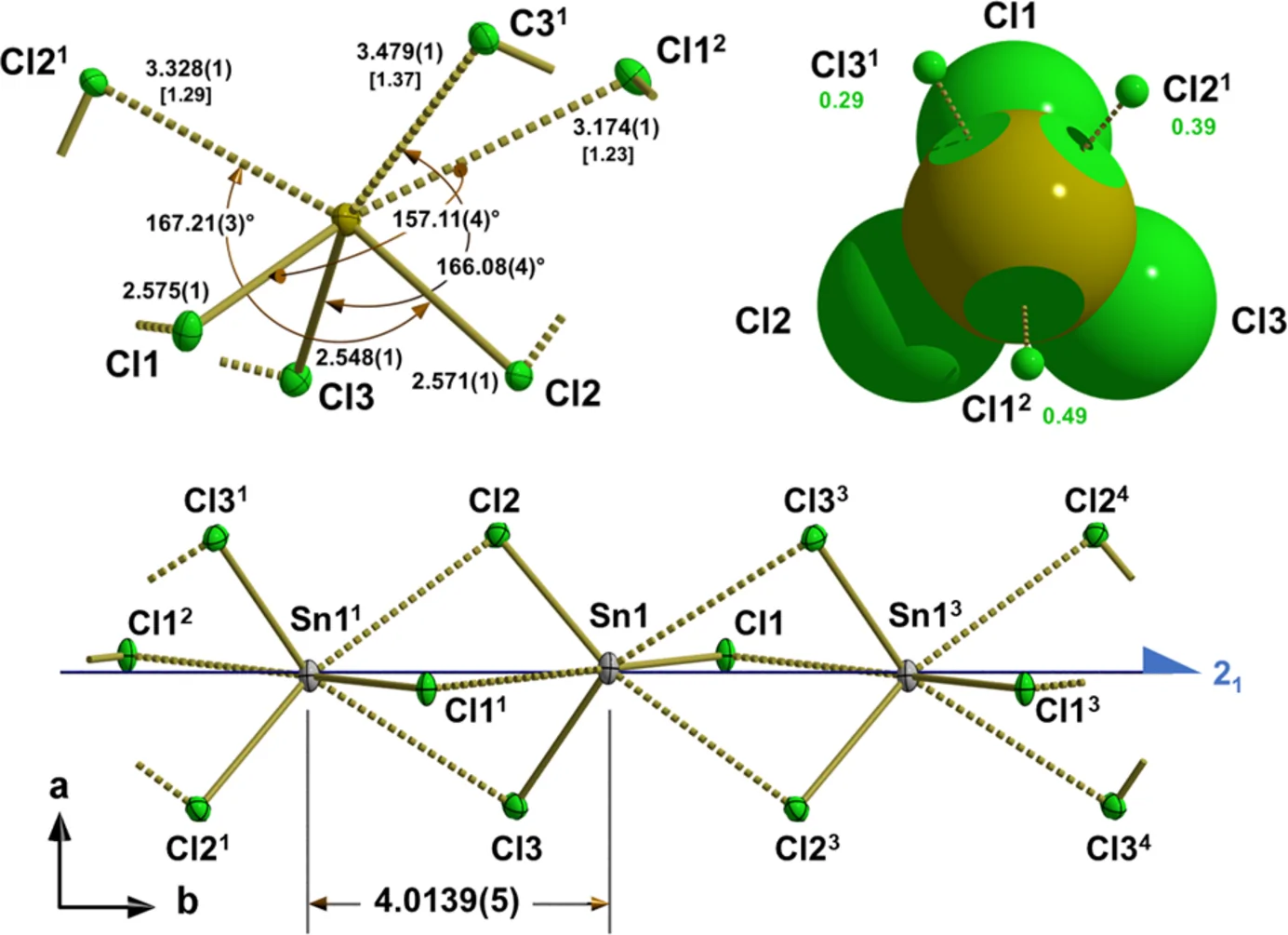
Different representation of the tetrel bonds linking the [SnCl_3_]^−^ ions. (above, left) ball-and-stick model of the tin environment with geometric parameters (Å, °) and asymmetry parameters *Q* (values in square brackets) characterizing the tetrel bonds, (above, right) space-filling model visualizing the inter-penetration of the van der Waals radii of tin and chlorine as result of the tetrel bond formation and inter­penetration indices *p* (green values), (below) side-view on the twofold screw axis causing the tetrel-bond formation with the distance (Å) between two neighbouring tin atoms. Symmetry codes used to generate equivalent atoms: (1) 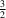 − *x*, 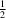 + *y*, 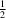 − *z*; (2) 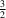 − *x*, −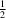 + *y*, 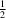 − *z*.

Based on the structural parameters observed in present case, the [SnCl_3_]^−^ ion must, unambiguously, be classified in the second group. A look inside the crystal (Fig. 2 ▸) reveals that the anions are arranged in rows along the *b* axis with alternating orientations caused by a twofold crystallographic screw axis in the *b-*axis direction (Fig. 3 ▸), while the cations are stacked in a staggered arrangement along the *a* axis. In the case of the anions, the inter­actions result in three *tetrel bonds* (Fig. 3 ▸*a*, Table 2 ▸). With an asymmetry quotient *Q* = *d*(Sn⋯Cl)/d(Sn—Cl) = 1.365 (Cl3), 1.294 (Cl2) and 1.233 (Cl1), these 3*c*–4*e* bonds are highly asymmetric, causing the *tpy*, 3_0_ configuration (Schröder *et al.*, 2024 ▸), of the first coordination sphere being extended to a highly distorted octa­hedral, 3_3_-aaa configuration. Similarly, the inter-penetration indices *ρ* (Echeverriá & Alvarez, 2023 ▸) using the van der Waals radii [*r*_vdW_(Cl) = 1.75 Å and *r*_vdW_(Sn) = 2.17 Å; Cordero *et al.*, 2008 ▸] and the covalent radii [*r*_cov_(Cl) = 1.02 Å and *r*_cov_(Sn) = 1.39 Å; Mantina *et al.*, 2009 ▸] support this concept. Furthermore, the calculation of bond valences, BS, according to Brese & O’Keeffe (1991 ▸) [*R_ij_* = 2.36, *b* = 0.37] shows that the inter­molecular Sn⋯Cl distances contribute only slightly [0.23] to the calculated bond valence sum [BVS_cal_ = 1.96] of the divalent tin atom.

**Figure 4:**
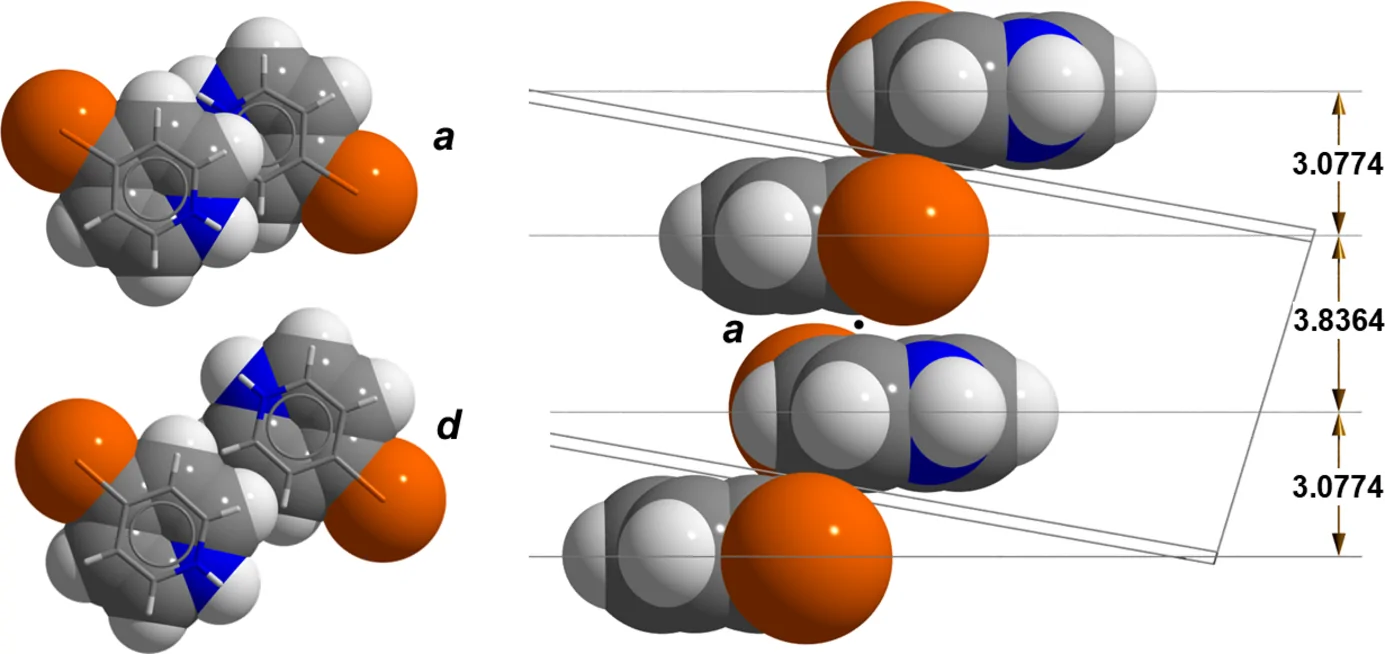
Different representations of the cation stacking. (left) Space-filling model with stick model overlayed viewed perpendicular to the least-squares planes showing the extend of the overlap as resulting from the different centres of symmetry in Wyckoff positions *a*, and *d.* (right) Space-filling model of the stacking sequence in side-view with distances [Å] between the least-squares planes; from the two different centres of symmetry (black dots) responsible for the stacking sequence only the one at (1/2,1/2,1/2), Wyckoff position *a* is visible.

The 4-bromo­pyridinium ions are stacked exactly parallel to one another, as they are flanked on both sides by crystallographic centres of symmetry at (0,0,0; Wyckhoff letter: *a*) and (1/2,0,0; Wyckhoff letter: *d*). The overlap of the pyridinium units, and thus the π–π inter­action, is limited to the nitro­gen atoms and two neighbouring carbon atoms (Fig. 4 ▸*a*). In this context, the extent of the overlap correlates with the distances between the least-squares planes defined by the ring atoms of neighbouring mol­ecules (Fig. 4 ▸*b*): in the case of the centres of symmetry at *a*, the distance is significantly shorter (3.08 Å) and the overlap smaller than in the case of the centers of symmetry at *d* (3.84 Å) where the overlap is larger.

In addition to their contribution to the tetrel bonds, the chlorine atoms, Cl2 and Cl3, are involved into hydrogen bonds with the hydrogen atom attached to the nitro­gen atom of the 4-bromo­pyridinium ion (Table 3 ▸). The donor–acceptor distances are quite long, as the hydrogen bond is bifurcated.

## Synthesis and crystallization

Stannous chloride (0.146 g (0.77 mmol); Sigma-Aldrich) was dissolved in approximately 10 ml of water together with 0.150 g (0.77 mmol) of 4-bromo­pyridine hydro­chloride. Upon slow evaporation of the aqueous solutions at room temperature, the title compound crystallized out in the form of colourless blocks. Yield: 0.256 g (86% of the theoretical yield).

## Refinement

Crystal data, data collection and structure refinement details are summarized in Table 4 ▸. The maximum and minimum residual electron density peaks of 1.40 and −0.91 e Å^−3^, respectively, were located 1.99 and 1.97 Å from the H6 and Sn1 atoms, respectively.

## Supplementary Material

Crystal structure: contains datablock(s) I. DOI: 10.1107/S2414314626008771/tk4130sup1.cif

Structure factors: contains datablock(s) I. DOI: 10.1107/S2414314626008771/tk4130Isup2.hkl

CCDC reference: 2582901

Additional supporting information:  crystallographic information; 3D view; checkCIF report

## Figures and Tables

**Table 1:** Selected geometric parameters (Å, °)

Sn1—Cl3	2.5475 (12)	Sn1—Cl1	2.5745 (11)
Sn1—Cl2	2.5711 (12)		
			
Cl3—Sn1—Cl2	90.73 (4)	Cl2—Sn1—Cl1	88.30 (4)
Cl3—Sn1—Cl1	88.32 (4)		

**Table 2:** Geometric details (Å), asymmetry parameters *Q*, bond valences BV and inter­penetration indices *p* characterizing the tetrel bonds

*X*/*Y*	*d*(Sn—*X*)	*d*(Sn⋯*Y*)	〈(*X*—Sn⋯*Y*)	*Q*	BV(Sn⋯*Y*)	BV(Sn—*X*)	*p*(Sn⋯*Y*)
Cl1/Cl1^2^	2.575 (1)	3.174 (3)	167.21 (3)°	1.23	0.05	0.60	0.49
Cl2/Cl2^1^	2.571 (1)	3.328 (3)	166.08 (4)°	1.29	0.07	0.57	0.39
Cl3/Cl3^1^	2.548 (1)	3.479 (3)	157.11 (4)°	1.37	0.11	0.56	0.29
Sum					0.23	1.73	

Symmetry codes: (1) 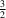 − *x*, 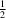 − *y*, 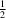 − *z*; (2) 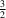 − *x*, −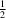 − *y*, 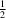 − *z*.

**Table 3:** Hydrogen-bond geometry (Å, °)

*D*—H⋯*A*	*D*—H	H⋯*A*	*D*⋯*A*	*D*—H⋯*A*
N1—H1⋯Cl2^i^	0.88	2.77	3.357 (4)	125
N1—H1⋯Cl3	0.88	2.48	3.255 (4)	147

Symmetry code: (i) 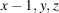.

**Table 4:** Experimental details

Crystal data	
Chemical formula	(C_5_H_5_BrN)[SnCl_3_]
*M*_r_	384.05
Crystal system, space group	Monoclinic, *P*2_1_/*n*
Temperature (K)	100
*a*, *b*, *c* (Å)	7.2468 (2), 8.0253 (3), 17.7393 (6)
β (°)	96.834 (2)
*V* (Å^3^)	1024.35 (6)
*Z*	4
Radiation type	Mo *K*α
μ (mm^−1^)	7.12
Crystal size (mm)	0.15 × 0.12 × 0.04

Data collection	
Diffractometer	Bruker APEXII CCD
Absorption correction	Multi-scan (*SADABS*; Krause *et al.*, 2015 ▸)
*T*_min_, *T*_max_	0.415, 0.774
No. of measured, independent and observed [*I* > 2σ(*I*)] reflections	97897, 2562, 2231
*R*_int_	0.079
(sin θ/λ)_max_ (Å^−1^)	0.668

Refinement	
*R*[*F*^2^ > 2σ(*F*^2^)], *wR*(*F*^2^), *S*	0.030, 0.073, 1.28
No. of reflections	2562
No. of parameters	101
H-atom treatment	Only H-atom displacement parameters refined
Δρ_max_, Δρ_min_ (e Å^−3^)	1.40, −0.91

Computer programs: *APEX2* and *SAINT* (Bruker, 2009 ▸), *SHELXS97* (Sheldrick, 2008 ▸), *SHELXL2014/7* (Sheldrick, 2015 ▸), *DIAMOND* (Brandenburg, 2006 ▸) and *Mercury* (Macrae *et al.*, 2020 ▸) and *publCIF* (Westrip, 2010 ▸).

## full crystallographic data

### 4-Bromopyridinium trichloridostannate(II) Crystal data

**Table:**

(C_5_H_5_BrN)[SnCl_3_]	*F*(000) = 712
*M**_r_* = 384.05	*D*_x_ = 2.490 Mg m^−^^3^
Monoclinic, *P*2_1_/*n*	Mo *K*α radiation, λ = 0.71073 Å
*a* = 7.2468 (2) Å	Cell parameters from 9904 reflections
*b* = 8.0253 (3) Å	θ = 2.3–27.5°
*c* = 17.7393 (6) Å	µ = 7.12 mm^−^^1^
β = 96.834 (2)°	*T* = 100 K
*V* = 1024.35 (6) Å^3^	Bloc, colourless
*Z* = 4	0.15 × 0.12 × 0.04 mm

### 4-Bromopyridinium trichloridostannate(II) Data collection

**Table:**

Bruker APEXII CCD diffractometer	2231 reflections with *I* > 2σ(*I*)
φ and ω scans	*R*_int_ = 0.079
Absorption correction: multi-scan (SADABS; Krause *et al.*, 2015)	θ_max_ = 28.3°, θ_min_ = 2.8°
*T*_min_ = 0.415, *T*_max_ = 0.774	*h* = −9→9
97897 measured reflections	*k* = −10→10
2562 independent reflections	*l* = −23→23

### 4-Bromopyridinium trichloridostannate(II) Refinement

**Table:**

Refinement on *F*^2^	Primary atom site location: structure-invariant direct methods
Least-squares matrix: full	Hydrogen site location: inferred from neighbouring sites
*R*[*F*^2^ > 2σ(*F*^2^)] = 0.030	Only H-atom displacement parameters refined
*wR*(*F*^2^) = 0.073	*w* = 1/[σ^2^(*F*_o_^2^) + (0.0158*P*)^2^ + 6.4943*P*] where *P* = (*F*_o_^2^ + 2*F*_c_^2^)/3
*S* = 1.28	(Δ/σ)_max_ = 0.001
2562 reflections	Δρ_max_ = 1.40 e Å^−^^3^
101 parameters	Δρ_min_ = −0.91 e Å^−^^3^
0 restraints	

### 4-Bromopyridinium trichloridostannate(II) Special details

**Table:**

Geometry. All esds (except the esd in the dihedral angle between two l.s. planes) are estimated using the full covariance matrix. The cell esds are taken into account individually in the estimation of esds in distances, angles and torsion angles; correlations between esds in cell parameters are only used when they are defined by crystal symmetry. An approximate (isotropic) treatment of cell esds is used for estimating esds involving l.s. planes.
Refinement. The H atoms were refined in idealized geometries and allowed to ride on the parent carbon [*d*(C—H) = 0.95 Å] and nitrogen atoms [*d*(N—H) = 0.88 Å] with one common isotropic temperature factor.

### 4-Bromopyridinium trichloridostannate(II) Fractional atomic coordinates and isotropic or equivalent isotropic displacement parameters (Å^2^)

**Table:**

	*x*	*y*	*z*	*U*_iso_*/*U*_eq_	
Sn1	0.75694 (5)	0.43421 (4)	0.24976 (2)	0.01293 (9)	
Cl1	0.78133 (17)	0.63009 (14)	0.36546 (6)	0.0161 (2)	
Cl2	1.00513 (16)	0.24850 (15)	0.32359 (6)	0.0159 (2)	
Cl3	0.50030 (16)	0.27775 (15)	0.30640 (7)	0.0180 (2)	
Br1	0.24791 (7)	1.05162 (6)	0.52167 (3)	0.01847 (12)	
N1	0.2791 (6)	0.5686 (5)	0.3868 (2)	0.0182 (8)	
H1	0.2893	0.4752	0.3616	0.034 (8)*	
C4	0.2545 (6)	0.8551 (6)	0.4651 (3)	0.0137 (9)	
C5	0.2871 (7)	0.8616 (6)	0.3896 (3)	0.0174 (10)	
H5	0.2998	0.9652	0.3648	0.034 (8)*	
C6	0.3003 (7)	0.7125 (6)	0.3517 (3)	0.0177 (10)	
H6	0.3248	0.7128	0.3003	0.034 (8)*	
C2	0.2428 (8)	0.5598 (7)	0.4590 (3)	0.0220 (11)	
H2	0.2260	0.4545	0.4817	0.034 (8)*	
C3	0.2299 (8)	0.7035 (6)	0.5002 (3)	0.0204 (11)	
H3	0.2048	0.6993	0.5515	0.034 (8)*	

### 4-Bromopyridinium trichloridostannate(II) Atomic displacement parameters (Å^2^)

**Table:**

	*U*^11^	*U*^22^	*U*^33^	*U*^12^	*U*^13^	*U*^23^
Sn1	0.01820 (16)	0.00819 (14)	0.01255 (14)	0.00055 (13)	0.00242 (11)	−0.00069 (12)
Cl1	0.0255 (6)	0.0100 (5)	0.0128 (5)	0.0000 (4)	0.0024 (4)	−0.0014 (4)
Cl2	0.0160 (5)	0.0147 (5)	0.0171 (5)	0.0021 (4)	0.0020 (4)	−0.0018 (4)
Cl3	0.0166 (5)	0.0159 (5)	0.0223 (6)	−0.0020 (4)	0.0052 (4)	−0.0052 (4)
Br1	0.0200 (2)	0.0136 (2)	0.0216 (2)	−0.00019 (19)	0.00136 (18)	−0.00675 (19)
N1	0.023 (2)	0.015 (2)	0.0169 (19)	0.0013 (18)	0.0026 (16)	−0.0060 (17)
C4	0.013 (2)	0.012 (2)	0.016 (2)	0.0024 (17)	0.0010 (17)	−0.0023 (18)
C5	0.022 (3)	0.013 (2)	0.017 (2)	0.0022 (19)	0.0036 (19)	−0.0010 (19)
C6	0.019 (2)	0.021 (3)	0.014 (2)	−0.0008 (19)	0.0030 (18)	−0.0022 (19)
C2	0.037 (3)	0.012 (2)	0.018 (2)	−0.002 (2)	0.005 (2)	0.000 (2)
C3	0.031 (3)	0.021 (3)	0.011 (2)	0.001 (2)	0.008 (2)	−0.0009 (19)

### 4-Bromopyridinium trichloridostannate(II) Geometric parameters (Å, º)

**Table:**

Sn1—Cl3	2.5475 (12)	C4—C5	1.388 (7)
Sn1—Cl2	2.5711 (12)	C5—C6	1.381 (7)
Sn1—Cl1	2.5745 (11)	C5—H5	0.9500
Br1—C4	1.873 (5)	C6—H6	0.9500
N1—C6	1.330 (7)	C2—C3	1.374 (7)
N1—C2	1.340 (6)	C2—H2	0.9500
N1—H1	0.8800	C3—H3	0.9500
C4—C3	1.387 (7)		
			
Cl3—Sn1—Cl2	90.73 (4)	C4—C5—H5	121.1
Cl3—Sn1—Cl1	88.32 (4)	N1—C6—C5	120.4 (5)
Cl2—Sn1—Cl1	88.30 (4)	N1—C6—H6	119.8
C6—N1—C2	122.7 (4)	C5—C6—H6	119.8
C6—N1—H1	118.7	N1—C2—C3	119.8 (5)
C2—N1—H1	118.7	N1—C2—H2	120.1
C3—C4—C5	120.8 (4)	C3—C2—H2	120.1
C3—C4—Br1	119.0 (4)	C2—C3—C4	118.5 (4)
C5—C4—Br1	120.2 (4)	C2—C3—H3	120.8
C6—C5—C4	117.8 (5)	C4—C3—H3	120.8
C6—C5—H5	121.1		

### 4-Bromopyridinium trichloridostannate(II) Hydrogen-bond geometry (Å, º)

**Table:**

*D*—H···*A*	*D*—H	H···*A*	*D*···*A*	*D*—H···*A*
N1—H1···Cl2^i^	0.88	2.77	3.357 (4)	125
N1—H1···Cl3	0.88	2.48	3.255 (4)	147

Symmetry code: (i) *x*−1, *y*, *z*.
